# Synergistic Fermentation with Functional Microorganisms Improves Safety and Quality of Traditional Chinese Fermented Foods

**DOI:** 10.3390/foods12152892

**Published:** 2023-07-29

**Authors:** Jingya Fan, Guanyi Qu, Datao Wang, Jian Chen, Guocheng Du, Fang Fang

**Affiliations:** 1 Science Center for Future Foods, Jiangnan University, Wuxi 214122, China; 6210208012@stu.jiangnan.edu.cn (J.F.); 7220201016@stu.jiangnan.edu.cn (G.Q.); 7210201069@stu.jiangnan.edu.cn (D.W.); jchen@jiangnan.edu.cn (J.C.); gcdu@jiangnan.edu.cn (G.D.); 2 Key Laboratory of Industrial Biotechnology, Ministry of Education, School of Biotechnology, Jiangnan University, Wuxi 214122, China; 3 Engineering Research Center of Ministry of Education on Food Synthetic Biotechnology, Jiangnan University, Wuxi 214122, China; 4 Jiangsu Province Engineering Research Center of Food Synthetic Biotechnology, Jiangnan University, Wuxi 214122, China

**Keywords:** fermented foods, synergistic fermentation, functional microorganisms, food safety, aroma

## Abstract

Traditional fermented foods are favored by people around the world for their positive health and taste advantages. Many of the fermented foods, including Chinese traditional fermented foods, are produced through mixed-culture fermentation. Apart from reducing the formation of harmful compounds such as ethyl carbamate (EC) and biogenic amines (BAs) during food fermentation, it is also difficult to precisely control and regulate the fermentation process based on the control of environmental conditions alone, due to the complex microbiota and an unclarified fermentation mechanism. In this review, key microorganisms involved in Chinese fermented foods such as baijiu, soy sauce, and vinegar production are elaborated, and relations between microbial composition and the aroma or quality of food are discussed. This review focuses on the interpretation of functions and roles of beneficial (functional) microorganisms that participate in food fermentation and the discussion of the possibilities of the synergistic use of functional microorganisms to improve the safety and quality of Chinese fermented foods. Conducting work toward the isolation of beneficial microorganisms is a challenge for modern food fermentation technology. Thus, methods for the isolation and mutagenesis of functional microbial strains for synergistic food fermentation are summarized. Finally, the limitations and future prospects of the use of functional microorganisms in traditional Chinese fermented foods are reviewed. This review provides an overview of the applications of synergistic fermentation with functional microorganisms in the improvement of the safety or sensory qualities of fermented foods.

## 1. Introduction

**Figure 1:**
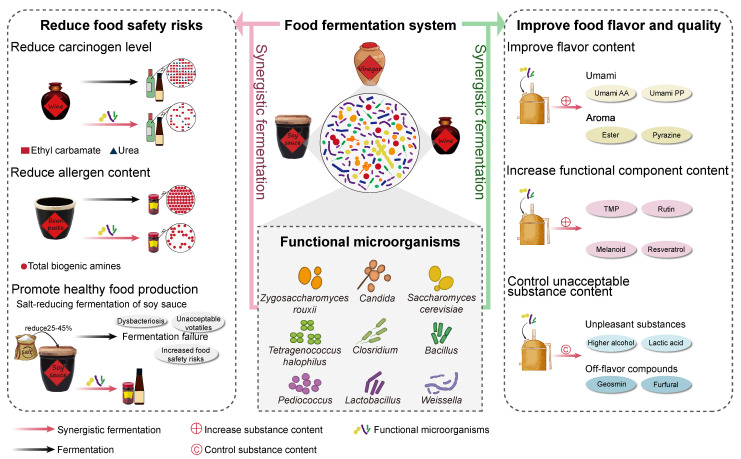
Synergistic fermentation with functional microorganisms improves safety and quality of traditional fermented foods.

Fermented foods have unique flavors and functional components and are generally produced through the growth and metabolism of a combined mixture of microorganisms. Baijiu, vinegar, soy sauce, paste, and pickles are essential compositions of traditional Chinese fermented foods. In the process of fermentation, microorganisms synthesize organic acids, alcohols, amino acids, esters, phenols, and other substances that confer special aroma and flavor to fermented foods [1,2,3,4,5]. Today, traditional food fermentation can be easily carried out by the addition of starters. However, problems such as batch variances, the formation of unwanted compounds, and the requirement of a long fermentation period are observed due to the mixed-culture fermentation style. Improvement in the flavor and quality of fermented foods through the optimization of the fermentation procedures has tended to be a bottleneck, with the increasing demand for safe, healthy, and functional food with improved quality by the consumers. Functional microorganisms play important roles in improving the safety and quality of fermented foods, due to their influences on both the microbial community and substance metabolisms during fermentation [1,6,7,8]. Synergistic fermentation is a practical strategy for the employment of functional microorganisms in the process of food fermentation [9,10,11,12]. It has gradually shown the potential capability to reduce the safety risk and improve the flavor and quality of fermented foods such as baijiu, soy sauce, and vinegar (Figure 1). The elucidation of the microbial community composition and succession patterns during the food fermentation process and disclosing their relations to food safety and quality are helpful in exploring the functional microorganisms and their main roles during food fermentation. The selection of functional microorganisms and uncovering the impact of synergistic fermentation with functional microorganisms on food safety and quality is helpful to provide a critical theoretical and practical basis for improving and enhancing the safety and quality of fermented foods.

## 2. Microbial Community and Functions of Food Microbiota in the Process of Food Fermentation

Microorganisms involved in food fermentation mainly come from natural starter cultures (e.g., leavening dough) or artificial starter cultures (e.g., jiuqu, koji, starter) and environments [13,14,15]. Generally, the microbial community composition is diverse and complex in traditional food fermentation systems. Yeast, filamentous fungi, lactic acid bacteria (LAB), acetic acid bacteria, *Bacillus*, and *Clostridium* are the main microorganisms involved in the traditional food fermentation process (Table 1). Although the food fermentation microflora is complex, diverse, and variable, key (core or dominant) microorganisms exist in individual stages. Key microorganisms play crucial roles in driving the fermentation process and regulating the synthesis and accumulation of beneficial components or other substances that affect the quality and flavor of fermented foods [2,16,17]. The characterization of the metabolisms of functional microorganisms in the process of food fermentation and microbial interactions helps to disclose the roles of functional microorganisms in fermentation and provides the theoretical basis for investigating the mechanisms of synergistic fermentation.

Baijiu is a popular Chinese spirit that is produced through solid-status fermentation, distillation, and years of aging [1]. Generally, the fermentation of baijiu takes 30–60 days according to different types of Chinese baijiu [2,13]. Materials for making Chinese baijiu are various. Sorghum, corn, wheat, rice, and glutinous rice are used to make strong-aroma baijiu, while sorghum is used to make jiang-flavor and light-flavor baijiu [2,13]. Qu is the starter used for baijiu fermentation, which comprises multiple microorganisms that come from muqu (the inoculum for making qu) and the environment. In the early stage of strong-aroma baijiu fermentation, *Saccharomyces*, *Issatchenkia*, *Rhizopus*, *Saccharomycopsis*, and *Aspergillus* are the dominant fungi, and *Bacillus* is the dominant bacteria [1]. Enzymes such as amylase produced by *Rhizopus*, *Saccharomycopsis*, and *Bacillus* [18] and protease and glucoamylase produced by *Aspergillus* can resolve or release starch and protein from raw materials and convert them into fermentable sugars and amino acids [2]. These substances provide both carbon and nitrogen sources and other elements for the growth and metabolism of microorganisms in the process of baijiu fermentation. *Bacillus subtilis* and *Bacillus licheniformis* in fermented grains (jiupei) synthesize functional components, i.e., pyrazines, that contribute to the unique flavor of baijiu [19,20]. The dominant fungi in the middle and late stages of strong-aroma baijiu fermentation include *Saccharomyces*, *Issatchenkia*, *Candida*, and *Hansenula* [2]. In this period, yeasts mainly carry out alcohol fermentation, providing important mellow substances and precursors to form ethyl esters in baijiu. In addition, *Saccharomyces cerevisiae* can synthesize higher alcohols, phenols, and other flavor substances [21] that are important volatiles for enhancing the mellowness and providing a unique aroma to baijiu [22]. Ester-producing yeasts such as *Hansenula*, *Candida*, *Pichia*, and *Brettanomyces* synthesize esterase to catalyze the formation of esters from their corresponding substrates [1,23]. Among them, *Hansenula*, *Candida*, and *Pichia* mainly contribute to the synthesis of acetate esters [24]. *Lactobacillus*, *Staphylococcus*, and *Pediococcus* are the dominant bacteria in the middle stage of strong-aroma baijiu fermentation [25]. These bacteria synthesize organic acids such as lactic acid, acetic acid, and butyric acid under anaerobic conditions. These acids are the primary organic acids and precursors for synthesizing corresponding esters in baijiu. With the production and accumulation of organic acids, the growth and metabolism of yeasts and acid-intolerant bacteria are inhibited. In the late stage of baijiu fermentation, *Lactobacillus* and *Lactobacillus acetotolerans* turn out to be the absolute dominant genus and species in the system [26,27]. Lactic acid and ethanol produced from the previous stage are catalyzed by esterases to form ethyl lactate, which is the crucial volatile in strong-flavor baijiu.

Chinese soy sauce (high-salt liquid-state) is a condiment made from plant-based materials (such as soybean and wheat) with high protein content. *Aspergillus oryzae* is inoculated to steam cooked materials to make koji (30 °C, 40–48 h), and the koji is then mixed with saline (18%) at a ratio of 1:2 (*w*:*v*) and fermented at 30 °C for 4–6 months [3,4]. The fermentation process for the production of high-salt liquid-state soy sauce consists of three main stages: the lactic acid fermentation stage, the ethanol fermentation stage, and the aging stage [28]. *Aspergillus oryzae* is the dominant fungus in the lactic acid fermentation stage. It produces hydrolases such as amylase, protease, and lipase to hydrolyze starch and protein and transform them into fermentable sugars, peptides, and amino acids, which allows the growth of other microorganisms and the synthesis of the main substances and flavor components in soy sauce [16]. In the lactic acid fermentation stage, LAB including *Weissella*, *Pediococcus, Tetragenococcus*, *Bacillus,* and *Staphylococcus* are the dominant bacteria in the moromi mash. They utilize substrates to synthesize organic acids such as lactic acid and acetic acid and produce peptidase to generate oligopeptides or amino acids from the hydrolysis of proteins [29]. With the synthesis and accumulation of organic acids in the lactic acid fermentation process, it turns into a suitable environment for the growth of yeast, and the ethanol fermentation stage begins. In the ethanol fermentation stage, salt-tolerant yeasts, mainly *Zygosaccharomyces rouxii*, synthesize the main volatiles of soy sauce such as ethanol, 4-ethyl guaiacol (4-EG), and pyrazines and contribute to the formation of esters [3,7,12]. In addition, yeasts can promote the synthesis of glycerol, succinic acid, and other substances by synergistic fermentation with other microorganisms, which helps improve the quality and flavor of soy sauce [30]. Jiang (bean paste) is a fermented condiment with regional features. It is different in raw materials (broad bean, wheat flour, and chili) and fermentation processes from soy sauce [17,31,32]. The dominant bacteria in the process of Pixian doubanjiang (broad-bean paste) fermentation are *Bacillus*, *Lactobacillus*, *Staphylococcus*, and *Pseudomonas* [4,18]. The dominant fungi of the microbial community are *Candida*, *Aspergillus*, and *Z. rouxii* [17]. *Bacillus* usually has the capability in the production of amylase and protease [18]. *B. subtilis*, *B. licheniformis*, and *Bacillus pumilus* present high activities of peptidase and transaminase, which are of vital importance to shorten the fermentation period and improve the quality of the Pixian doubanjiang [16]. *Pseudomonas*, *Staphylococcus*, and *Aspergillus* are also important microorganisms involved in Pixian doubanjiang fermentation. They can degrade peptides and contribute to the formation of flavor-nitrogenous compounds [4,33]. *Pseudomonas* is found to be related to the formation of 3-methyl butyraldehyde, 2-methyl butyraldehyde, 5-methyl-2-phenyl-2-hexenal, and other essential volatiles in Pixian doubanjiang [33]. Yeasts may be related to synthesizing volatiles such as 2-ethylphenol and 4-ethyl-2-methoxyphenol in Pixian doubanjiang [6,34]. *Z. rouxii* is a functional yeast that contributes to the formation of ethanol and other volatiles such as phenylethyl alcohol, 3-methyl butanol, ethyl acetate, and phenethyl acetate in Pixian doubanjiang [35,36].

Chinese traditional vinegar is made from cereals (rice, sorghum, wheat bran, or malt) by mixed-culture and solid-state fermentation for about one month [37,38,39]. The fermentation process includes three stages: saccharification, alcoholic fermentation, and acetic acid fermentation. Among them, acetic acid fermentation is the critical stage that gives vinegar its unique flavor and brings nutritional function [8,40,41]. *Mucor*, *Absidia*, and *Aspergillus* are the core fungi in the saccharification stage [5,42,43]. They can synthesize amylase, lipase, and protease to hydrolyze starch, fat, and protein and convert them into small molecules such as glucose, fatty acids, and peptides, which provide nutrients for the growth and metabolism of other microorganisms during fermentation [44]. Yeasts involved in vinegar fermentation include *Saccharomyces*, *Saccharomycopsis*, and *Pichia* [42,43]. In the alcoholic fermentation stage, *S. cerevisiae* utilizes fermentable sugars to produce ethanol. This provides the essential precursor for the synthesis of acetic acid and contributes to the formation of alcohols and esters in vinegar [41,45]. *Saccharomycopsis* contributes to the formation of fermentable substances in the system by the secretion of amylase and protease [45]. LAB such as *Lactobacillus*, *Weissella*, *Pediococcus*, and *Leuconostoc* are the dominant bacteria in both the saccharification stage and alcohol fermentation stage [42,46,47]. LAB synthesize lactic acid and short-chain fatty acids and play an important role in softening the taste and buffering the irritation of vinegar [48,49]. LAB also synthesize proteases and aminopeptidases to promote protein hydrolysis and produce peptides and amino acids, enriching and enhancing the flavor of vinegar [42,50]. Acetoin is a precursor for synthesizing tetramethylpyrazine (TMP), a volatile that gives vinegar a unique flavor and function [49,51,52]. In the process of vinegar fermentation, *Lentilactobacillus buchneri*, *Limosilactobacillus reuteri*, *Levilactobacillus brevis*, and *Limosilactobacillus fermentum* are identified to produce enzymes that are responsible for synthesizing acetoin [53]. *Acetobacter* strains are the dominant bacteria in the acetic acid fermentation stage. They can produce acetic acid from ethanol and produce esters, amino acids, and other substances that contribute to the flavor of vinegar [5,46]. *Acetobacter pasteurianus* is the main bacterium involved in vinegar fermentation. This species can synthesize the enzyme that converts diacetyl to acetoin [46,53,54].

The formation of nutrients and quality-related components and the synthesis of flavor compounds in Chinese traditional fermented foods are closely related to the metabolisms of microorganisms such as *Aspergillus oryzae*, yeasts, LAB, and non-LAB bacteria. Recently, the roles and mechanisms of some of these microorganisms participating in mixed-culture food fermentation have been gradually disclosed, either through the characterization of their fermentation-related traits or by synergistic fermentation with them during food fermentation. This greatly aids the finding of efficient ways to regulate and control both the microbial community and microorganisms’ metabolisms during food fermentation.

**Table 1:** Function and roles of microorganisms involved in Chinese traditional food fermentation.

Figure		Role	Origin	References
Yeast	*Saccharomyces*	Synthesize alcohols	Baijiu	[1,2]
	*Saccharomyces cerevisiae*	Synthesize volatiles (higher alcohols, phenols)	Baijiu, vinegar	[1,2,41,45]
	*Issatchenkia*	Synthesize alcohols	Baijiu	[1,2]
	*Rhizopus*	Produce amylase	Baijiu	[1,18]
	*Saccharomycopsis*	Produce amylase and protease	Baijiu, vinegar	[1,18,44,45]
	*Hansenula*	Synthesize alcohols, produce esterase	Baijiu	[1,2,23,24]
	*Candida*	Synthesize alcohols, produce esterase	Baijiu	[1,2,23,24]
	*Pichia*	Produce esterase	Baijiu	[23,24]
	*Brettanomyces*	Produce esterase	Baijiu	[23]
	*Zygosaccharomyces rouxii*	Synthesize volatiles (ethanol, 4-EG, pyrazines, phenylethyl alcohol, 3-methyl butanol, ethyl acetate, and phenethyl acetate)	Soy sauce, jiang (bean paste)	[3,7,12,35,36]
Filamentous fungi	*Aspergillus*	Produce protease, glucoamylase, amylase, and lipase	Baijiu, soy sauce, jiang (bean paste)	[1,2,4,5,16,33,44]
	*Mucor*	Produce amylase, lipase, and protease	Vinegar	[5,42,43,44]
	*Absidia*	Produce amylase, lipase, and protease	Vinegar	[5,42,43,44]
Functional bacteria	*Clostridium*	Synthesize butyric acid and acetic acid	Baijiu	[11,55]
	*Bacillus*	Synthesize organic acids, produce amylase, peptidase, and protease	Baijiu, soy sauce, jiang (bean paste)	[1,4,18,29]
	*Bacillus subtilis*	Synthesize pyrazines, produce peptidase and transaminase	Baijiu, jiang (bean paste)	[16,19,20]
	*Bacillus licheniformis*	Synthesize pyrazines, produce peptidase and transaminase	Baijiu, jiang (bean paste)	[16,19,20]
	*Bacillus pumilus*	Produce peptidase and transaminase	Jiang (bean paste)	[16]
	*Staphylococcus*	Synthesize organic acids, produce peptidase	Baijiu, soy sauce	[25,29]
	*Tetragenococcus*	Synthesize organic acids, produce peptidase	Soy sauce	[29]
	*Pseudomonas*	Synthesize volatiles (3-methyl butyraldehyde, 2-methyl butyraldehyde, and 5-methyl-2-phenyl-2-hexenal), degrade peptides	Jiang (bean paste)	[4,33]
	*Acetobacter*	Synthesize acetic acid, amino acids, esters	Vinegar	[5,46]
	*Acetobacter pasteurianus*	Produce the enzyme that converts diacetyl to acetoin	Vinegar	[46,53,54]
LAB	*Lactobacillus*	Synthesize organic acids, produce peptidase, proteases, and aminopeptidases	Baijiu, vinegar	[25,42,46,47,48,49,50]
	*Levilactobacillus brevis*	Produce enzymes that are responsible for synthesizing acetoin	Vinegar	[53]
	*Limosilactobacillus fermentum*	Produce enzymes that are responsible for synthesizing acetoin	Vinegar	[53]
	*Pediococcus*	Synthesize organic acids, produce peptidase, proteases, and aminopeptidases	Baijiu, soy sauce, vinegar	[25,29,42,46,47,48,49,50]
	*Weissella*	Synthesize organic acids, produce peptidase, proteases, and aminopeptidases	Soy sauce, vinegar	[29,42,46,47,48,49,50]
	*Leuconostoc*	Synthesize organic acids, produce proteases and aminopeptidases	Vinegar	[42,46,47,48,49,50]

## 3. Functions of Synergistic Fermentation in Maintaining Food Safety

In the process of food fermentation, microorganisms synthesize important components and flavor substances through biochemical metabolisms. Some microorganisms may synthesize allergic, carcinogenic, and unpleasant biohazard substances or their precursors during food fermentation [56,57,58,59]. These biohazard substances may form during food manufacture, such as fermentation, filtration, and distillation, resulting in increased food safety risks. This potentially raises the risk issues of food safety. However, it is difficult to control and regulate the metabolism of a single stain or multiple microorganisms in a mixed-culture system, and genetically engineered strains are neither allowed to be used nor acceptable for food production. Thus, the development of methods to decrease the content of harmful substances and their precursors during food fermentation is important to reduce the risk of food safety issues. Ethyl carbamate (EC, group 2A carcinogen) and biogenic amines (BAs) are biohazard compounds produced through microbial metabolisms during food fermentation [60,61]. The mechanisms of synthesis and accumulation of EC and its precursors have been clarified in the production process of baijiu, huangjiu, and soy sauce. It is mainly related to the insufficient metabolism of nitrogen compounds such as urea and arginine [56,62]. The formation of BAs in fermented foods is generally related to the decarboxylation of amino acids by bacteria (LAB mainly) involved in fermentation [63]. Synergistic fermentation with functional microorganisms has shown excellent effect in the reduction and control of EC and BAs in fermented foods with nearly no alterations in the fermentation process and food flavor and quality (Table 2). Strategies of using functional microorganisms to reduce and control the formation of EC or its precursors have been developed and successfully applied to the production of baijiu, huangjiu, and soy sauce on an industrial scale [9,10,64,65]. The amount of BAs in fermented foods can also be reduced by synergistic fermentation with strains that have low/no amino acid decarboxylase activity [66].

The main precursors of EC in baijiu are urea and citrulline [67,68,69]. Urea in baijiu may come from raw materials or the arginine metabolism of yeasts during fermentation [70]. Baijiu is produced through mixed-culture fermentation. It is challenging to efficiently regulate the synthesis of urea using a single strain or the engineered strain that produces no or low urea during baijiu fermentation. Interestingly, synergistic fermentation with urease (hydrolysis of urea into ammonia, carbon dioxide, and water) producers significantly reduces the synthesis and accumulation of urea, ultimately reducing EC content in baijiu [71]. *Bacillus amyloliquefaciens* JP21 is a urease producer isolated from fermented grains. Synergistic fermentation with *Bacillus amyloliquefaciens* JP21 can reduce urea and EC content in baijiu by 50.05% and 30.16%, respectively, during strong-flavor baijiu fermentation [9]. Moreover, the employment of *B*. *amyloliquefaciens* JP21 in baijiu fermentation has no significant effect on volatile composition in baijiu [68]. *B. licheniformis* DX530 is another bacterium isolated from fermented grains of strong-flavor baijiu, it can degrade EC precursors urea and citrulline. Synergistic fermentation with *B. licheniformis* DX530 decreases citrulline and urea in fermented grains by 11% and 10%, respectively, and finally EC in baijiu is reduced by 16% [72]. *Lysinibacillus sphaericus* MT33 is a urease producer isolated from fermented grains of sesame-flavor baijiu. Synergistic fermentation with *L*. *sphaericus* MT33 reduces urea and EC contents in fermented grains by 28.15% and 41.77%, respectively. Moreover, synergistic fermentation with *L*. *sphaericus* MT33 significantly increases the contents of volatiles such as isopentyl valerate, ethyl octanoate, and butyl caproate in baijiu [10]. Thus, synergistic fermentation with functional bacteria during fermentation can successfully decrease the content of biohazard compounds in baijiu, efficiently reducing food safety risks.

EC is also detected in soy sauce, especially high-salt liquid-state (HSL) soy sauce. The main precursor of EC in HSL soy sauce is citrulline [62], which is synthesized by bacteria that have the ADI (arginine deiminase) pathway during fermentation [73]. In the lactic acid fermentation stage, LAB, mainly *Pediococcus acidilactici*, can utilize arginine to produce citrulline through the ADI pathway [73]. In the ethanol fermentation stage, dominant bacteria including *Staphylococcus*, *T. halophilus,* and *B. amyloliquefaciens* produce citrulline through the ADI pathway, and the accumulation of citrulline is enhanced by increased cell membrane permeability due to the effect of free fatty acids and ethanol [57]. *B. amyloliquefaciens* JY06, a salt-tolerant bacterium isolated from soy sauce moromi, can efficiently utilize arginine and hardly accumulate citrulline. The synergistic fermentation of soy sauce with *B. amyloliquefaciens* JY06 significantly reduces citrulline and EC by 80.9% and 82.5%, respectively. Moreover, the flavor of soy sauce is also improved by synergistic fermentation with *B. amyloliquefaciens* JY06 [65].

Urea and citrulline are the precursors of EC in huangjiu. *S. cerevisiae* synthesizes urea via the urea cycle, while LAB use arginine as the substrate to synthesize citrulline through the ADI pathway during huangjiu fermentation [64,74]. Although *S. cerevisiae* can consume urea, it preferentially utilizes preferred nitrogen sources such as glutamate due to nitrogen catabolite repression (NCR), resulting in the accumulation of the non-preferred nitrogen source, urea [75,76]. Currently, reducing and controlling EC content in huangjiu is mainly accomplished through synergistic fermentation with microorganisms that produce less urea or are capable of utilizing citrulline [64,77]. These strains are obtained either by the high-throughput screening of wild-type strains or a combination of adaptive evolution or mutation breeding with high-throughput screening. *S. cerevisiae* N14 with low urea-producing capacity is a mutant of *S. cerevisiae* N85 obtained by adaptive evolution. Synergistic fermentation with *S. cerevisiae* N14 can reduce urea by 16.8%, with no significant differences in the content of amino acids in a simulated huangjiu fermentation system [64]. Another mutant, *S. cerevisiae* 5–11C, is less sensitive to nitrogen catabolite repression, and it can reduce the accumulation of urea by efficiently utilizing urea. The employment of *S. cerevisiae* 5–11C in the simulated fermentation system reduces urea in huangjiu by 50.6%, compared with that of using the wild-type strain [78]. *Lactobacillus brevis* 2–34 is a huangjiu isolate that can efficiently utilize citrulline [79]. Synergistic fermentation with *L. brevis* 2–34 has no obvious changes in the flavor of huangjiu, and the contents of citrulline and EC in huangjiu are reduced by 58.2% and 29.6%, respectively [77]. Synergistic fermentation with functional microorganisms isolated from food fermentation systems has shown great potential in the reduction of biohazards such as EC and BAs in the process of baijiu, soy sauce, huangjiu, and paste production. This provides practical references for the regulation of the metabolism of specific substances in a complex mixed-culture fermentation process without affecting the quality and flavor of fermented foods.

**Table 2:** Reduction of biohazard compounds in fermented foods by synergistic fermentation with functional microorganisms.

Functional Microorganisms	Function	Reduction of Biohazard Compounds		Food	Reference
		Compounds	Reduction Rate (%)		
*Bacillus amyloliquefaciens* JP21	Produce urease	Urea	50.05	Baijiu	[9]
		EC	30.16		
*Bacillus licheniformis* DX530	Degrade urea and citrulline	Citrulline	11	Baijiu	[68]
		Urea	10		
		EC	16		
*Bacillus amyloliquefaciens* JY06	Control the synthesis of citrulline	Citrulline	80.9	Soy sauce	[65]
		EC	82.5		
*Lysinibacillus sphaericus* MT33	Produce urease	Urea	28.15	Baijiu	[10]
		EC	41.77		
*Saccharomyces cerevisiae* N14	Low urea-producing capacity	Urea	16.8	Huangjiu	[64]
*Saccharomyces cerevisiae* 5–11C	Utilize urea	Urea	50.6	Huangjiu	[78]
*Lactobacillus brevis* 2–34	Utilize citrulline	Citrulline	58.2	Huangjiu	[79]
		EC	29.6		
*Lactobacillus plantarum* HM24	Degrade BAs	Total BAs	35.79	Soybean paste	[80]
*Staphylococcus piscifermentans* CGMCC 18053, *Zygosaccharomyces rouxii* CICC 1417, and *Torulopsis candida* CICC 1019	Degrade BAs	Total BAs	63.25	Soy sauce	[81]

## 4. Improvement in Food Flavor and Quality by Synergistic Fermentation with Functional Microorganisms

The enhancement or regulation of the synthesis of volatiles, nutrients, or functional substances in the fermentation process is an efficient way for improving the quality and flavor of fermented foods [82,83,84]. Current studies have clarified the functions of a few microorganisms in food fermentation and proposed some correlations between metabolites and microbial or environmental factors in mixed-culture fermentation [85,86,87,88,89]. However, it is still challenging to control the composition and metabolisms of microorganisms at a stable level in different batches of food fermentation. Meanwhile, it is also difficult to precisely regulate the synthesis of beneficial components (such as nutrients and volatiles) and unacceptable components (off-flavor compounds and unpleasant substances) during food fermentation through simple optimization of the fermentation process [90]. In addition, process optimization may cause potential problems such as extended production time and increasing costs. Functional microorganisms usually are the dominant species in food fermentation systems. They are reasonably easy to isolate and cultivate and have advantages in adaption to the environment of food fermentation [82,91,92]. Therefore, there is a promising prospect for synergistic fermentation with functional microorganisms in upgrading food fermentation technology, increasing economic efficiency by improving the utilization of raw materials, and regulating the synthesis of substances related to the quality and flavor of fermented foods.

For fermented foods, minor components also influence and determine the flavor and quality of food except for the major components [83]. Esters are the key flavor substances that impart unique aromas to baijiu. Previous studies disclose that ethyl acetate brings a fruity aroma to light-flavor baijiu [84], and ethyl caproate brings cellar fragrances and a fruity aroma to strong-flavor baijiu [93]. Direct addition of esterase during fermentation could be used for increasing the content of flavor esters in baijiu. However, this may not be practical for the underground solid-state fermentation of baijiu. Moreover, the addition of enzymes either in food or for food manufacture has to follow the regulations for using food additives. It has been demonstrated that synergistic fermentation with functional microorganisms that produce esterase or synthesis precursors of esters could increase the content of esters in baijiu. *S. cerevisiae* Y3401 and *Wickerhamomyces anomalus* Y3604 are two strains isolated from fermented grains of light-flavor baijiu. They are confirmed to be ethanol and ethyl esterase producers. Synergistic fermentation with a complex microbial community including these two yeasts significantly enhances the content of ethyl acetate in light-flavor baijiu. Consequently, the quality of light-flavor baijiu is improved with increased content of esters [94].

In mixed-culture fermentation, slight alterations in microbial composition or environmental conditions may bring the synthesis of off-flavor compounds or overproduction of volatiles that cause discomfort when present at a high level in food. Geosmin (GSM) is an earthy or musty off-flavor compound found in light-flavor baijiu with the contamination of *Streptomyces* in the process of making qu [58,95]. Studies showed that *S. cerevisiae*, *Pichia*, and *Bacillus* could effectively inhibit the growth of *Streptomyces* [96]. By employing a complex microbial community composed of *S. cerevisiae* LBM22005, *Bacillus* sp. LBM12033, *Pichia* sp. LBM22006, *Pichia* sp. LBM22007, *Pichia* sp. LBM22008, and *Pichia* sp. LBM22009 in the process of making qu, the content of geosmin in qu is reduced to 4.05 ± 0.86 μg·kg^−1^. This is 51.01% less than that in qu without synergistic fermentation with functional microorganisms [96]. Lactic acid is an essential organic acid in Chinese baijiu. However, excessive production of lactic acid during fermentation influences the synthesis of ethanol and affects the quality and flavor of baijiu [97]. Synergistic fermentation with functional microorganisms has shown positive results in reducing lactic acid accumulated in fermented grains during baijiu fermentation. Motile *Clostridium* species have been isolated from the pit mud of strong-flavor baijiu. Most of them present strong chemotaxis to lactic acid and the capability of utilizing lactic acid to produce butyric acid and acetic acid [11,55]. In a simulated strong-flavor baijiu fermentation system, a complex microbial community composed of a few strains of *Clostridium* is added to the pit mud. Lactic acid in fermented grains works as a chemokine that triggers *Clostridium* to migrate from pit mud to fermented grains and utilize lactic acid there, resulting in a 38.9% reduction in lactic acid and an increase in acetic acid and butyric acid in fermented grains [55]. It is demonstrated that the synergistic fermentation of Maotai-flavor baijiu with *Pichia* ATCC6258 could reduce lactic acid in fermented grains by 64.9%, while no influence on the ethanol content is observed [97]. Moreover, synergistic fermentation with *P. kudriavzevii* C-16 promotes the growth of *S. cerevisiae* C-3 in the presence of lactic acid and increases the utilization of lactic acid by 34.3% [98]. Higher alcohols are important flavor volatiles that may lead to drinking discomfort when present at a high level in spirits. Synergistic fermentation with *S. cerevisiae* jiangnan1# can reduce the content of higher alcohols by 25% in huangjiu [99], and synergistic fermentation with *Clostridium tyrobutyricum* ZY-4 can reduce the content of butanol by 30% in the fermented grains of strong-flavor baijiu [100].

Soy sauce is a condiment that mainly confers umami and saltiness tastes and a soy-sauce-like aroma to food. The types and contents of amino acids, peptides, and some volatiles are associated with the umami of soy sauce. It is confirmed that the synergistic fermentation of high-salt liquid-state soy sauce with functional microorganisms such as *Tetragenococcus halophilus*, *Z. rouxii*, and *Wickerhamiella versatilis* could increase the content of amino acid nitrogen and enhance the umami taste [12,101]. Furthermore, synergistic fermentation with *B. subtilis* D445A, which has the ability to produce γ-glutamyl transpeptidase, increases the content of glutamic acid (umami amino acid) in soy sauce [102]. Soy sauce aroma is mainly associated with volatiles such as 2-ethyl-4-hydroxy-5-methylfuran-3-one (HEMF), 4-EG, and phenylacetaldehyde [103]. And 1-octen-3-ol brings a mushroom aroma to soy sauce [101,104]. Synergistic fermentation with *Z. rouxii* and *T. halophilus* results in the increase in total volatiles, guaiacol, and 1-octen-3-ol in soy sauce moromi mash by 2.4 folds, 11.1 folds, and 8.9 folds, respectively [104]. Synergistic fermentation with *Starmerella etchellsii* CICIMY0600 results in a significant increase in main volatiles 4-EG and HEMF in soy sauce [12]. Interestingly, synergistic fermentation with *T. halophilus* CGMCC 3792, *Z. rouxii* CGMCC 3791, and *W. versatilis* CGMCC 3790 can increase the content of 1-octen-3-ol by 56.7% in soy sauce moromi. And this stimulates a significant increase in the contents of other volatiles such as 2-methyl-1-butanol, isoamyl acetate, benzaldehyde, and TMP [101].

Chinese traditional rice vinegar has more than thousands of volatiles and various bioactive substances, including amino acids, polyphenols, and TMP [105]. Except for being a vital flavor substance that brings nutty and soy sauce aroma to vinegar, TMP is a functional substance that promotes blood circulation, protects the liver, and improves immunity [20,41,106]. A positive correlation between the yield of acetoin (the precursor of TMP) and the ability of *Bacillus* strains to produce protease has been identified previously [107]. Thus, synergistic fermentation with *Bacillus mojavensis* B15 and *Bacillus methylotrophic* B6 in the process of vinegar fermentation increases the content of TMP in vinegar mash to 15.76 μg·g^−1^, which is 1.3 times higher than the control [108].

These studies demonstrate an efficient improvement in the flavor and quality of fermented foods by synergistic fermentation with functional microorganisms, through the increase in flavor compounds and functional components, and the reduction or elimination of unacceptable substances (Figure 1). To increase flavor compounds and functional components, functional microorganisms can produce enzymes associated with catalyzing the synthesis of flavor or functional substances, provide the precursors of flavor or functional substances, or utilize substrates to synthesize metabolites that improve food flavor and function. In addition, synergistic fermentation with functional microorganisms can reduce or eliminate unacceptable substances, since functional microorganisms can utilize unacceptable substances to produce metabolites with no negative influence on food flavor and quality, or can inhibit the growth of other microorganisms producing unacceptable substances.

## 5. Selection and Mutagenesis of Functional Microorganisms for Food Fermentation

**Figure 2:**
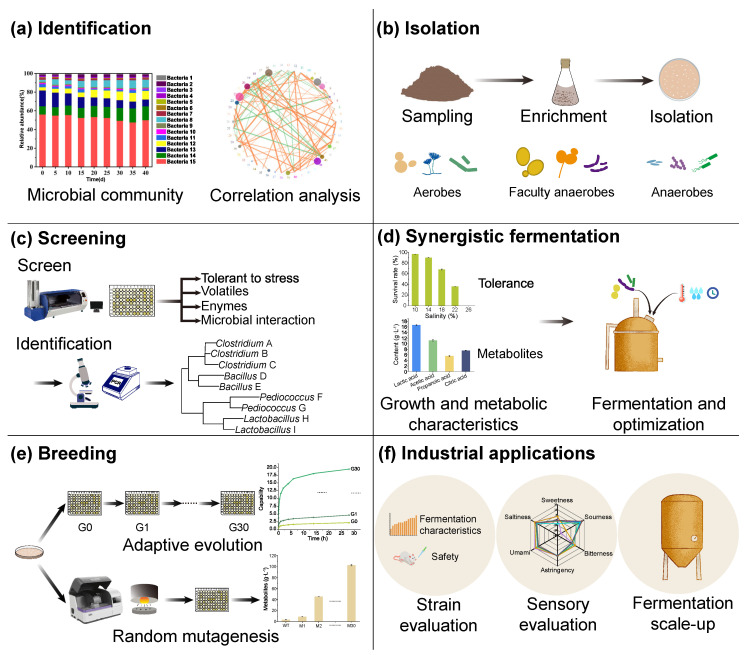
Selection and mutagenesis of functional microorganisms for synergistic fermentation.

Synergistic fermentation with functional microorganisms has become an effective measure and a promising technology to improve the safety and quality of fermented foods. The key to performing synergistic fermentation is to have functional microorganisms with suitable traits for food fermentation [82,83,94]. Exploring functional microorganisms and the characterization of their roles in food fermentation help to establish targets for isolating functional microorganisms [109,110]. Thus, the selection of functional microorganisms and mutagenesis of them with improved fermentation traits become an essential prerequisite for employing them in synergistic fermentation. Current methods for the selection and mutagenesis of functional microorganisms include random mutagenesis and adaptive evolution based on conditional selection, using the high-throughput operation platform (Figure 2).

### 5.1. Selection and Mutagenesis of Microorganisms to Reduce Biohazard Compounds in Fermented Foods

Functional strains used for food fermentation can be efficiently isolated and selected using selective media containing biohazard compounds combined with high-throughput screening. A selective medium supplemented with urea can be used for isolating strains that have the capability to degrade urea. *Staphylococcus saprophyticus* M39 is a strain obtained in this way, and it can effectively reduce the amount of both urea and EC during baijiu fermentation [91]. Using a selective medium supplemented with EC, *B. amyloliquefaciens* JP21, a strain that can reduce the content of urea and EC in baijiu during fermentation was obtained from fermented grains [68]. Random mutagenesis of microorganisms by using chemicals or physical treatment is an efficient and acceptable way to obtain mutants from wild-type strains for food fermentation. *T. halophilus* R23 is a soy sauce moromi isolate that can efficiently utilize arginine and accumulate low levels of citrulline under normal conditions [110]. A mutant strain *T. halophilus* 3-H9, obtained through ultraviolet (UV) and atmospheric and room-temperature plasma (ARTP) mutations, is more effective in reducing the accumulation of arginine and citrulline than the wild-type strain *T. halophilus* R23 in a simulated soy sauce fermentation system [109]. Moreover, mutants *B. amyloliquefaciens* C12 and E6 obtained through UV and ARTP mutations have better capability in the reduction of citrulline than the wild-type strain JY06. In comparison to JY06, synergistic fermentation with C12 and C6, citrulline, and EC in soy sauce can be reduced by 15.6% and 14.7%, 19.3%, and 13.1%, respectively [62].

Adaptive evolution is also a method to obtain mutants of functional microorganisms for food fermentation. Therefore, it is possible to breed mutants of functional strains that have better capability in degrading biohazard substances or their precursors than the wild-type strain. *S. cerevisiae* XZ-11 is a wild strain with low urea-production capacity isolated from the huangjiu fermentation system. To obtain strains with lower urea-producing capacity than XZ-11, continuous cultivation in a YNB medium containing 0.6 g·L^−1^ urea is used for the adaptive evolution of XZ-11. Eventually, three mutants of *S*. *cerevisiae* XZ-11 with decreased urea-production capability are obtained. Among them, mutant 4B is the most effective in reducing urea content in a simulated huangjiu fermentation system. With the employment of *S*. *cerevisiae* 4B, the reduction in urea is increased by 48.0% [64].

### 5.2. Selection and Mutagenesis of Microorganisms to Improve the Quality of Fermented Foods

Functional microorganisms used for improving the flavor and quality of various traditional fermented foods can be obtained through the selection and mutagenesis of microorganisms that are isolated from the food fermentation system. *B*. *subtilis* YHB0165, *B. subtilis* YHB0169, *B. subtilis* YHB0171, and *Bacillus megatherium* YHB0170 are beneficial bacteria isolated from the high-temperature daqu of jiang-flavor baijiu. Synergistic fermentation with them contributes to the formation of baijiu volatiles such as TMP, 2,3,5-trimethylpyrazine, and 2,6-dimethylpyrazine, as well as acetic acid, butyric acid, and other aroma compounds [111]. *B. velezensis* FZB42, an isolate of daqu for making strong-flavor baijiu, can significantly increase the content of volatiles including acids, alcohols, and ethyl hexanoate in baijiu [112].

For the non-targeted selection of functional microorganisms with excellent fermentation traits for food fermentation, a combined mutation and breeding method is effective. *S. cerevisiae* S48, a yeast with stable genetic properties and low acetaldehyde production capacity is obtained by ARTP mutation, for brewing beer. With the employment of *S. cerevisiae* S48 in beer fermentation, the acetaldehyde content in beer is reduced by 23.69%, and the beer has an enhanced harmonious flavor [113,114]. *S. cerevisiae* with lower acetaldehyde production capacity can be obtained efficiently using the combination of mutation and adaptive evolution [115]. Moreover, a mutant of *A. oryzae* H34 with 145.6% increased protease activity is successfully obtained using ARTP mutation integrated with a flow cytometry high-throughput operation platform. The employment of this mutant in fermentation significantly increases the content of total nitrogen, amino acid nitrogen, and flavored organic acids in soy sauce [116].

## 6. Conclusions

Many Chinese traditional fermented foods are produced through mixed-culture fermentation mainly in a solid or semi-solid state. The formation of nutrients and quality-related components and the synthesis of flavor compounds in fermented foods are closely related to microbial metabolisms. The succession of the microbial community during food fermentation is relatively controllable but can be easily disturbed by contamination or environment alterations, resulting in fermentation disorder, rising safety risks (formation of biohazard compounds), or defects in quality and aroma (decrease in flavor volatiles and increase in unacceptable volatiles). Synergistic fermentation with functional microorganisms has shown great advantages in the reduction and control of the formation of biohazard or unacceptable compounds during food fermentation, as well as enhancing the flavor or contents of functional components in traditional fermented foods. The high-throughput isolation of functional microorganisms from food fermentation systems and the direct evolution and mutagenesis of microorganisms for improved traits have great potential in finding proper strains to enable a stable and controllable fermentation process for the production of safe, tasty, and healthy fermented foods.

## 7. Future Prospects

Traditional food fermentation requires a fine microbiota to produce fermented foods with stable quality and a nice flavor. The control and regulation of microbiota and microbial metabolisms during food fermentation can be aided at a certain stage with the employment of functional microorganisms. Different from the fermentation using a pure strain, the population of functional microorganisms in the traditional food fermentation system is independent of the inoculation level. For the industrial production of fermented foods, strains that are about to be used as functional microorganisms need to be a food-grade (evaluated by whole-genome sequencing and animal trials) or GRAS (generally regarded as safe) strain. In addition, the employment of functional microorganisms for food fermentation shall not affect the growth and metabolism of other key microorganisms for food fermentation, nor shall they have negative influences on food quality and flavor. Using simplified microbiota, i.e., synthetic microbiota comprising core microorganisms, is expected to achieve stable, efficient, and safe production of traditional fermented foods. Thus, the identification of functional microorganisms and disclosure of their effects on food fermentation both provide a good reference for finding and selecting core microorganisms (synthetic microbiota) for controllable food fermentation in the future.
